# Synthesis of Heterotelechelic Poly(*N*‐ethylglycine) Polymers: from Polypeptoid Lipid Conjugates to Protein‐Polymer Conjugates

**DOI:** 10.1002/marc.202500422

**Published:** 2025-07-16

**Authors:** Ewout Buijs, Zlata Nagorna, Joachim F. R. Van Guyse, Matthias Barz

**Affiliations:** ^1^ Leiden Academic Centre for Drug Research (LACDR) Leiden University Leiden The Netherlands

**Keywords:** NCA polymerization, poly(N‐ethylglycine), polymer‐lipid conjugates, polypeptoids, protein‐polymer conjugates

## Abstract

The need for alternative hydrophilic polymers to polyethylene glycol (PEG) has intensified due to increasing concerns about immunogenicity and hypersensitivity reactions. In this work, we report the synthesis of well‐defined heterotelechelic poly‐*N*‐ethylglycine (p*N*EtGly) via acid‐catalyzed ring‐opening polymerization of *N*‐substituted N‐carboxyanhydrides with a degree of polymerization from 25 to 400. The Leuchs synthesis was modified and optimized for the preparation of high‐purity *N*‐ethylglycine NCA monomers, enabling controlled polymerization with the use of organic acid catalysts. The resulting p*N*EtGly have low polydispersity values (Ð < 1.05) and quantitative end‐group fidelity, enabling the synthesis of well‐defined polymer–lipid and polymer–protein conjugates. A palmitamide‐functionalized p*N*EtGly demonstrated near‐quantitative conversion to polymer–lipid conjugates. Furthermore, maleimide‐functionalized p*N*EtGly was conjugated to human serum albumin (HSA) via thiol–maleimide coupling, forming a protein–polymer conjugate with high purity. These results establish p*N*EtGly as another interesting hydrophilic polymer for biomedical applications, particularly in lipid‐based drug delivery and bioconjugation strategies.

## Graphical abstract

Defined for conjugation. We report the synthesis of heterotelechelic poly(*N*‐ethylglycine) (p*N*EtGly) via acid‐catalyzed polymerization of high‐purity NCA monomers. The resulting polymers exhibit excellent end‐group fidelity and enable well‐defined, high‐purity protein and lipid conjugates. Thiol–maleimide coupling to HSA and near‐quantitative palmitamide lipid conjugation highlight pNEtGly as a robust, PEG‐free platform for next‐generation drug delivery strategies.

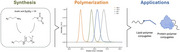

## 1 Introduction

Hydrophilic polymers are able to modulate the pharmacokinetics of therapeutics such as lipid‐based nanoparticles, e.g., liposomes, lipid nanoparticles (LNPs) or solid lipid nanoparticles (SLPs), proteins/peptides, and other small molecule drugs [1, 2, 3]. The conjugation of polymers can increase the size of therapeutic entities and create protein‐resistant surfaces by forming hydrating and entropic energy barriers, in addition to their steric properties, which further prevent aggregation [4, 5, 6]. These characteristics contribute to an improved pharmacokinetic profile by reducing opsonization and extending circulation time compared to unmodified particles, therefore, they are also referred to as stealth polymers [6]. First introduced in the early 1970s by Davis and coworkers, who conjugated polyethylene glycol (PEG) to therapeutic proteins (PEGylation) and observed reduced immunogenicity and increased circulation time [7]. Ever since these observations, PEG has been at the forefront of the field of drug delivery, leading to 38 FDA‐approved drugs by 2024 [8]. PEG's success can be mainly attributed to its high‐water solubility, biocompatibility, GMP grade production, and excellent control during polymerization. This includes precise control over molecular weights, complete end‐group fidelity, and the synthesis of polymers with narrow molecular weight distribution featuring a polydispersity below 1.1 [9]. However, its use in therapeutic settings faces rising concerns regarding PEG‐related immunogenicity, especially since the widespread use of PEGylated pharmaceuticals following the rollout of COVID‐19 vaccinations [10]. These concerns relate to the immune responses following from induced or pre‐existing anti‐PEG antibodies in patients, exerting effects, including hypersensitive reactions and accelerated blood clearance (ABC) phenomena [11, 12]. Therefore, the development of alternative polymers with comparable physicochemical properties and improved biocompatibility is crucial for the next generation of polymer conjugates [13, 14].

Polypeptoids, a class of polymers that feature an aliphatic polyamide backbone with substitution on the nitrogen atoms, have emerged as one of the most promising polymers in this regard [15, 16, 17]. The most notable stealth polymer in this class is polysarcosine (pSar), based on the endogenous amino acid sarcosine (N‐methyl glycine) [18, 19, 20]. Having very similar properties to PEG, which include its hydrophilicity, non‐ionic nature, and hydrogen bond acceptor properties [4]. However, in contrast to PEG, the amino acid sarcosine is an endogenous molecule taking part in glycine metabolism [16, 21]. This endogenous origin appears to reduce immunogenic responses and enhance biocompatibility [22]. Furthermore, variations in polymer design enable the fine‐tuning of physicochemical properties, offering flexibility that is advantageous for application‐driven systems [23, 24]. Changing the nitrogen substitution on polypeptoids without compromising the ‘Whiteside’ rules for protein resistance leaves little room for modification [25]. The most straightforward alternative *N*‐substitution for water‐soluble polypeptoids is towards the ethyl group, as longer aliphatic chains display thermoresponsive solution behavior or are completely insoluble in water [26, 27]. For this reason, poly(*N*‐ethylglycine) (p*N*EtGly) is an interesting alternative to pSar in the class of hydrophilic polymers for biomedical applications. The ethyl group introduces a slight increase in hydrophobicity and steric bulk compared to the methyl side chain of pSar, which may enhance resistance to hydrolysis under harsh conditions such as extreme pH or elevated temperatures. This structure–stability relationship mirrors observations in poly(2‐oxazolines), where replacing the methyl side chain in poly(2‐methyl‐2‐oxazoline) (PMeOx) with an ethyl group in poly(2‐ethyl‐2‐oxazoline) (PEtOx) leads to improved stability in acidic and thermal environments [28, 29]. Additionally, polypeptoids such as pSar and p*N*EtGly are generally considered chemically robust under acidic and reductive conditions, due to the absence of labile functional groups on the side chain and backbone [30]. Furthermore, *N*‐alkylation of amino acids is an effective way to increase resistance against proteases, potentially improving the overall stealth properties [31, 32].

Like pSar, p*N*EtGly may be applied in a wide range of pharmaceutical products where a stealth effect is required. These systems range from lipid nanoparticles (LNPs), solid lipid nanoparticles (SLPs), Polyion Complex Micelles (PICMs), protein/peptide conjugates, and drug conjugates [33, 34, 35, 36, 37, 38, 39]. To this date, no high molecular weight *N*‐ethylglycine polymers with low dispersities (Ð<1.1), monomodal distributions, and full end‐group fidelity have been synthesized [26, 40, 41, 42, 43]. If alternative hydrophilic polymers aim to replace PEG, the quality in polymer dispersity and end‐group fidelity should not be compromised. In general, polypeptoid‐based medium and high molecular weight polymers can be synthesized via ring opening polymerization (ROP) of *N*‐substituted *N*‐carboxyanhydrides (NNCAs) [44]. The success and control of NNCAs polymerizations are closely tied to the purity of the combined components in the process, which include the monomer, initiator, solvent(s), and the catalyst [45]. Additionally, the kinetics of the ring opening reactions are highly dependent on the *N*‐substitutions of the NCA [46]. Therefore, the successful synthesis of probability‐driven living polymerizations of p*N*EtGly is a multifactorial challenge that involves thorough purification in every step along the way, with additional optimization of the polymerization process itself.

**FIGURE 1:**
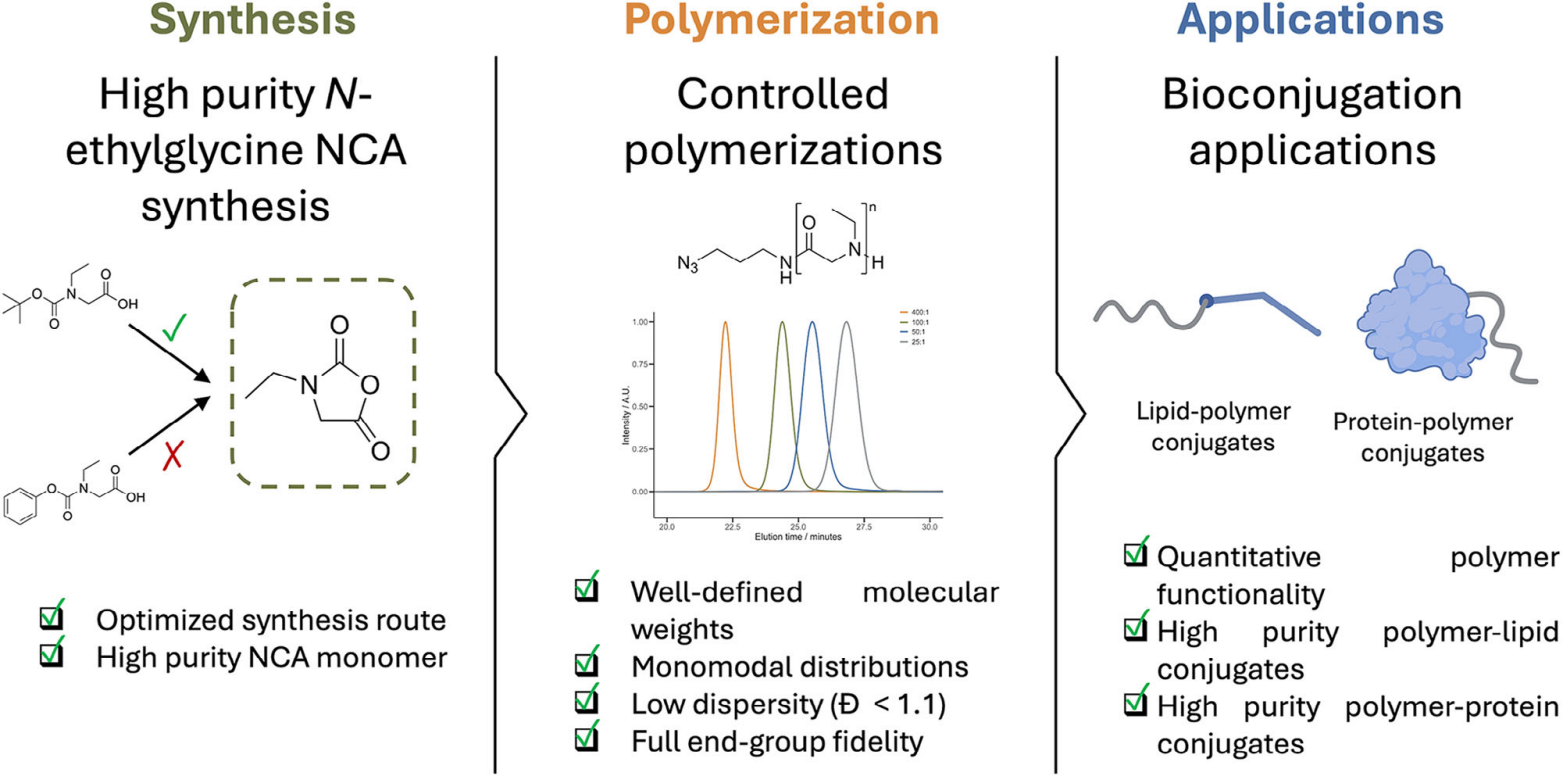
Overview of the synthesis, characterization, and bioconjugation applications of heterotelechelic poly(*N*‐ethylglycine) for drug delivery. The synthesis of high‐purity *N*‐ethylglycine N‐carboxyanhydride (NCA), controlled polymerization to well‐defined polymers, and subsequent application to lipid–polymer and protein–polymer conjugates are conceptually illustrated.

This work highlights the synthesis of p*N*EtGly via ring opening polymerization of NNCAs monomers with acetic acid catalysis. We report conditions for the controlled polymerization up to a degree of polymerization of 400 that lead to high molecular weights and quantitative heterotelechelic end‐group functionality. Additionally, we showcase possible applications in drug delivery for poly(*N*‐ethylglycine) by synthesizing polymer‐lipid and polymer‐protein conjugates (Figure 1).

## 2 Results and Discussion

### 2.1 Monomer Synthesis

**FIGURE 2:**
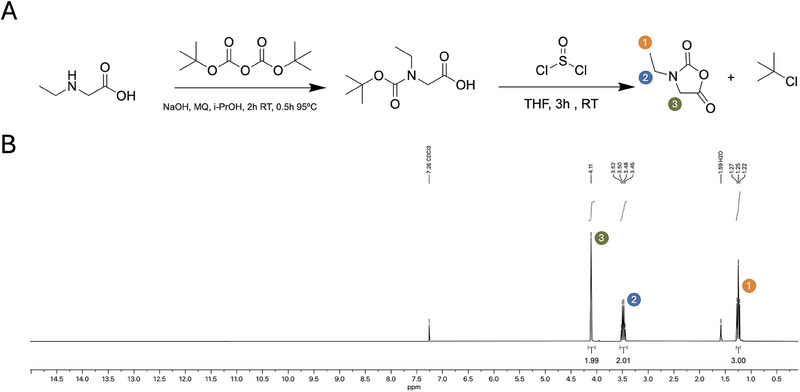
Synthesis and characterization of *N*‐ethylglycine N‐carboxyanhydride (NCA). (A) Synthetic scheme for the preparation of *N*‐ethylglycine NCA. (B) ^1^H NMR spectrum of the N‐ethylglycine NCA with corresponding integration values and peak assignments.

The *N*‐ethylglycine NCA monomer was synthesized via a method based on the Leuchs synthesis [46, 47, 48]. While the established pathway is based on Benzylchloroformate (Cbz) protective group, our research involved screening multiple protecting groups and halogenating agents [26]. We tested both Cbz and tert‐butyloxycarbonyl (BOC) (Figures S3 and S4). Despite vigorous purification by distillation, Cbz failed to yield NNCA in the highest purity (Figure S5). The combination of the BOC protection group and halogenation with thionyl chloride resulted in pure NCA after workup (Figure 2A). Since the main by product for this specific synthesis route is tert‐Butyl chloride, with a boiling point of 51°C, it can be easily removed under reduced pressure [49]. In contrast to the other common amine protection groups Cbz, which will form benzyl chloride with a boiling point of 179°C [50]. Therefore, purification of *N*‐ethylglycine NCA via distillation can conveniently be performed with the BOC protective group with reasonable efforts. Unlike most (N)NCAs, which are solid at room temperature, *N*‐ethylglycine NCA is obtained in a liquid form. As a result, standard purification methods commonly used for NCA monomers, such as sublimation or recrystallization are not suitable. Instead, we achieved high purity *N*‐ethylglycine NCA by applying vacuum distillation as introduced by Sisido et al. [51]. At a pressure of <0.1 millibar, the boiling point of the NCA is approximately 80°C. This allows us to perform distillation in a suitable temperature range, since our findings suggest that exposure to high temperatures for extended times leads to the polymerization of the crude product with the remaining impurities. Although no major impurities were detected in the crude NCA by ^1^H NMR spectroscopy (Figure S6), prolonged exposure to elevated temperatures may activate trace nucleophilic impurities, allowing them to initiate ring‐opening polymerization. Habraken et al. have shown that N‐carboxyanhydride monomers can polymerize more rapidly under reduced pressure and elevated temperatures, conditions present during distillation [52]. Moreover, NCAs are known to be thermally unstable and can decompose under such conditions [47]. As a result, the combined effects of vacuum and heat during distillation likely contribute to both premature NCA consumption and depletion of trace impurities. This process was accompanied by a visual change in the distillate from a slightly yellow to a clear liquid. ^1^H and ^13^C NMR analysis of the purified NCA correlates with all the theoretical and previously reported spectra of *N*‐ethylglycine NCA (Figures S7 and S8) [26]. Based on the NMR results, a very high purity >99% product is obtained (Figure 2B). Although the obtained purity is consistently high, the overall yields are comparatively low (∼14‐23%), varying with each batch. This reduction in yield is attributed to the previously discussed polymerization side reactions occurring under distillation conditions during purification. Nonetheless, in comparison to previously reported methods, this synthesis route improves purity significantly as judged by NMR [26].

### 2.2 Polymer Synthesis

**FIGURE 3:**
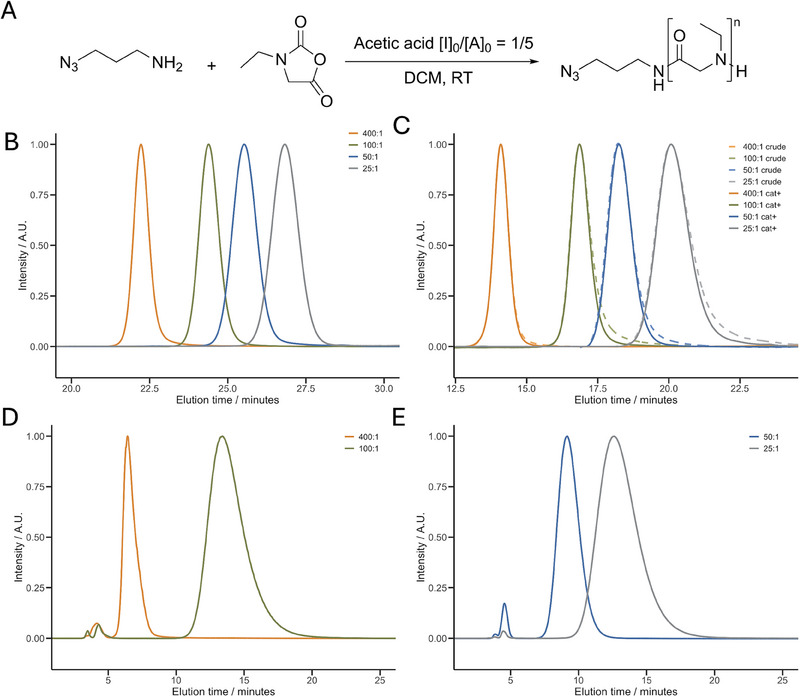
Characterization of poly(*N*‐ethylglycine) (p*N*EtGly). (A) Reaction scheme illustrating the initiator and conditions used for the polymerization of N‐ethylglycine. (B) UV_230nm_ SEC traces in HFIP of p*N*EtGly at different [M]_0_/[I]_0_ ratios with fixed [I]_0_/[AA]_0_ ratio of 1/5. (C) RI SEC traces in DMF of pNEtGly at different [M]_0_/[I]_0_ ratios with fixed [I]_0_/[AA]_0_ ratio of 1/5. (D) UV_220nm_ CEC‐traces in 0.5mM phosphate‐buffer at different [M]_0_/[I]_0_/[AA]_0_ ratios with fixed [I]_0_/[AA]_0_ ratio of 1/5. (E) UV_220nm_ CEC‐traces in 2mM phosphate‐buffer at different [M]_0_/[I]_0_/[AA]_0_ ratios with fixed [I]_0_/[AA]_0_ ratio of 1/5.

It is well‐established that the controlled‐living nature of NCA ring‐opening polymerizations is highly dependent on the purity of the monomers, solvents and initiators [53]. Moreover, optimization of the polymerization conditions plays a critical role in achieving controlled polymerization, significantly impacting parameters such as polydispersity, average molecular weight, and end‐group fidelity [45, 54]. Our polymerization reactions were all carried out in anhydrous DCM as this solvent provides good solubility for the *N*‐ethylglycine NCA and the p*N*EtGly, which has one of the fastest ROP kinetics and can be easily purified [54]. To enable a modular post‐polymerization modification of p*N*EtGly polymers, NCAs were initiated with 3‐azidopropanamine, which can be employed in azide‐alkyne coupling reactions post polymerization on the α‐end of the polypeptoid. The degrees of polymerization (DP) were set by the [M]_0_/[I]_0_ ratios between 25 to 400, which are significantly above the DPs reported before by Fetsch et al. in 2011 [26]. The polymers obtained under these conditions exhibited lower than calculated molecular weights, loss of end‐group fidelity, and a polydispersity above 1.1 (Table 1 & Figure S13). Cation exchange chromatography (CEC) can be used to separate polymers by charge, which in the case of p*N*EtGly polymers corresponds to end group integrity (secondary amine) or can identify water initiation (carboxylic acid α‐terminus) [45]. When the degree of polymerization exceeds 100, the occurrence of side reactions becomes evident as clearly depicted by the end‐group fidelity (AUC of the cationic fraction), despite rigorous purification of all components (Table 1). To improve the situation, the mechanism of the NNCA polymerization needs to be considered. The kinetics for the normal amine (NAM) ring opening polymerizations are limited by three major energy barriers: 1) the nucleophilic addition at the 5‐carbonyl group, 2) the ring opening of the NNCA and 3) the decarboxylation [54, 55]. In our case, the ethyl group at the amine position of *N*‐ethylglycine NCA introduces a more sterically hindered transition state and hinders the nucleophilic attack (Figure S14). This observation is consistent with the kinetic data reported by Fetsch et al., who demonstrated that the polymerization of *N*‐ethylglycine NCA proceeds significantly slower than that of sarcosine NCA, with apparent rate constants (k_p_^app^) of 7.41 and 305 × 10^−3^ L·mol^−1^·s^−1^ in benzonitrile, respectively [26]. To increase polymerization kinetics, we applied weak organic acids as introduced by Bamford et al. and recently revisited by Wang et al. and by us [45, 54, 56]. Therefore, acetic acid was added in a fixed [initiator]_0_/[acetic acid]_0_ ratio of 1/5 (Figure 3A). Under these conditions, we observed polymerizations of *N*‐ethylglycine in a quasi‐living manner, with [NCA]_0_/[Initiator]_0_ ranging from of 25 to 400 (Table 1). Size exclusion chromatography (SEC) in HFIP of the acid‐catalyzed polymers exhibited sharp and symmetrical peaks for all molecular weights, indicating a Poisson‐like distribution of molecular weights as reflected in the low dispersity values of Ð < 1.05 (Table 1 and Figure 3B). Therefore, this work offers a clear advancement over previously reported p*N*EtGly polymers [26, 40, 41, 42, 43]. Nevertheless, a careful analysis of SEC plots in DMF depicts a minimal low molecular weight tailing, suggesting that there is a two‐step initiation or termination process still present, either induced by slow initiation by residual impurities, or other mechanistic complexities yet to be fully elucidated (Figure 3C) [45]. Additionally, the theoretical and calculated molecular weights are in line with the calculated molecular weights, highlighting the controlled polymerization (Table 1). The polymers synthesized in the presence of the acetic acid exhibit end group fidelities from 99.3% to 94.9% (Table 1 and Figure 3D,E). Nonetheless, these polymers can be further purified via preparative cationic ion‐exchange chromatography. This method can be applied to remove the remaining neutral impurities quantitatively. The area under the curve for the cationic fraction remains similar within the standard‐deviation after preparative ion‐exchange, especially for crude polymers with initial cationic fractions of >99%. The slight low molecular weight tailing that can be observed in the DMF SEC traces, however, is removed as highlighted by the crude p*N*EtGly plots (Figure 3C). This leads to an overall improvement in polymer quality, as reflected by a further decrease in polydispersity values. Remarkably, these polymers are close to the theoretical limit (Đ $=\;1+\frac{1}{\mathrm{Degr}\mathrm{ee}\;\mathrm{of}\;\mathrm{poly}\mathrm{meri}\mathrm{zati}\mathrm{on}}$) (Table 1). Therefore, the removal of neutral impurities by preparative ion‐exchange arises as a robust strategy for enhancing pNEtGly (and other polypept(o)ides) quality. Upon establishing the synthesis of p*N*EtGly polymers with quantitative end‐group functionality and narrow dispersity, these well‐defined polymers serve as a versatile platform for the next generation of polymer–lipid and polymer–protein conjugates. Their controlled synthesis and high end‐group integrity enable efficient and selective post‐polymerization modifications, making them suitable for a wide range of bioconjugation strategies.

**TABLE 1:** Properties of various poly(N‐ethylglycine) products.

	Crude polymers					Purified polymers				
[M]_0_/[I]_0_/[AA]_0_	M_n_ (kg mol^−1^)_HFIP_ ^a^	M_n_ (kg mol^−1^)_DMF_ ^b^	Ð_HFIP_ ^c^	Ð_DMF_ ^b^	%AUC^c^	M_n_ (kg mol^−1^)_HFIP_ ^a^	M_n_ (kg mol^−1^)_DMF_ ^b^	Ð_HFIP_ ^c^	Ð_DMF_ ^b^	%AUC^d)^
25/1/0	1.6	1.4	1.12	1.11	85.3	—	—	—	—	—
50/1/0	2.8	3.0	1.13	1.13	81.8	—	—	—	—	—
100/1/0	4.6	3.6	1.10	1.10	78.9	—	—	—	—	—
200/1/0	6.5	4.4	1.14	1.16	77.8	—	—	—	—	—
25/1/5	1.3	1.5	1.03	1.07	99.3	1.7	1.6	1.02	1.05	quant.
50/1/5	2.9	3.2	1.04	1.06	95.3	2.9	3.2	1.03	1.04	99
100/1/5	5.3	5.4	1.03	1.05	98.4	6.7	5.8	1.02	1.03	99
400/1/5	21.8	25.8	1.06	1.09	94.9	24.4	27.5	1.04	1.09	99

^a^ Determined from pSar standards.

^b^ Determined from the DMF SEC according to the PEG standards.

^c^ Determined from PMMA standards.

^d^ Determined by analytical cation‐exchange chromatography.

### 2.3 Poly(*N‐*ethylglycine) Lipid‐Conjugates

**FIGURE 4:**
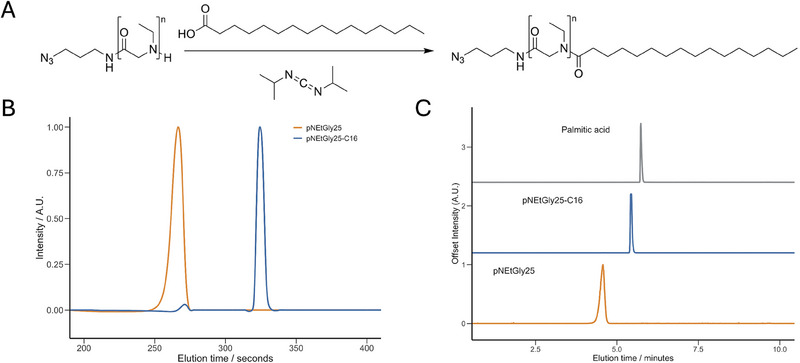
Polymer‐lipid conjugates from poly(*N*‐ethylglycine) and palmatic acid. (A) Reaction scheme highlighting the coupling of *poly(N*‐ethylgycine) homopolymer with palmitic acid to form palmitimade‐p*N*EtGly. (B) UPLC UV traces at 215nm of free cationic poly(*N*‐ethylgycine)_25_ versus the polymer‐lipid (16:0, PA) conjugate. (C) UPLC ELSD signals comparison between free polymer, polymer‐lipid conjugate (16:0, PA), and palmitic acid.

With the rise of LNP formulations for nucleic acid delivery, polymer‐lipid conjugates became crucial components in practically all lipid‐based nanoparticles [57, 58, 59, 60]. Therefore, a functionalization of pNEtGly to generate a polymer‐lipid conjugate enables a convenient pathway to alternatives for PEGylated lipids [61, 62]. To serve as a viable alternative, the polymer must meet the quality standards of PEG. Particularly, ω end‐group fidelity and accessibility are essential. If quantitative ω end‐group fidelity is absent, extensive purification is needed, which results in lower yields. Following confirmation of quantitative end‐group fidelity of the poly(*N*‐ethylglycine) product after preparative ion‐exchange chromatography (Table 1), a coupling reaction was carried out between poly(*N*‐ethylglycine) (25:1) and palmitic acid (16:0, PA) to generate the poly(*N*‐ethylglycine)‐palmitamide polymer‐lipid conjugate (Figure 4A). Post‐reaction purification was limited to precipitation and dialysis, reflecting the high efficiency and selectivity of the coupling process when high quality p*N*EtGly with full end‐group fidelity is used. Analysis of the final product via UPLC highlights near quantitative conversion to the lipid‐polymer conjugated product. Integration of the UV_220nm_ highlights >97% of the compound consists of the polymer‐lipid conjugate (Figure 4B). This can be concluded from the shift in elution time on the UPLC column (C18), highlighting the increased hydrophobicity stemming from the palmitic acid's aliphatic chains. The remaining fraction homo‐polymer can be attributed to the incomplete coupling with DIC, or to the residual neutral fraction, unable to participate in the coupling reaction. Analysis of the ELSD signal across the full UPLC run indicates that the polymer–lipid product was obtained with a final purity > 99% (Figure 4C) [63]. This is consistent with the purity levels reported for DMG‐PEG2K, a benchmark PEGylated lipid excipient used in lipid nanoparticle‐based mRNA delivery systems [64]. Finally, ^1^H DOSY NMR indicates a single diffusing species in which signals of pNEtGly and the terminal carbon from the palmitic group are included (0.85 ppm) (Figure S16). The secondary diffusion species observed in the DOSY measurement is attributed to the corresponding solvents DMSO‐d6 and D_2_O. These results underscore that high‐purity p*N*EtGly with quantitative ω‐end group fidelity enables near‐quantitative conversion to the corresponding polymer–lipid conjugate. Furthermore, the final product can be obtained with very high purity, as confirmed by UPLC and DOSY NMR analysis, following a simple work‐up procedure.

### 2.4 Poly(*N‐*ethylglycine) Protein Conjugates

**FIGURE 5:**
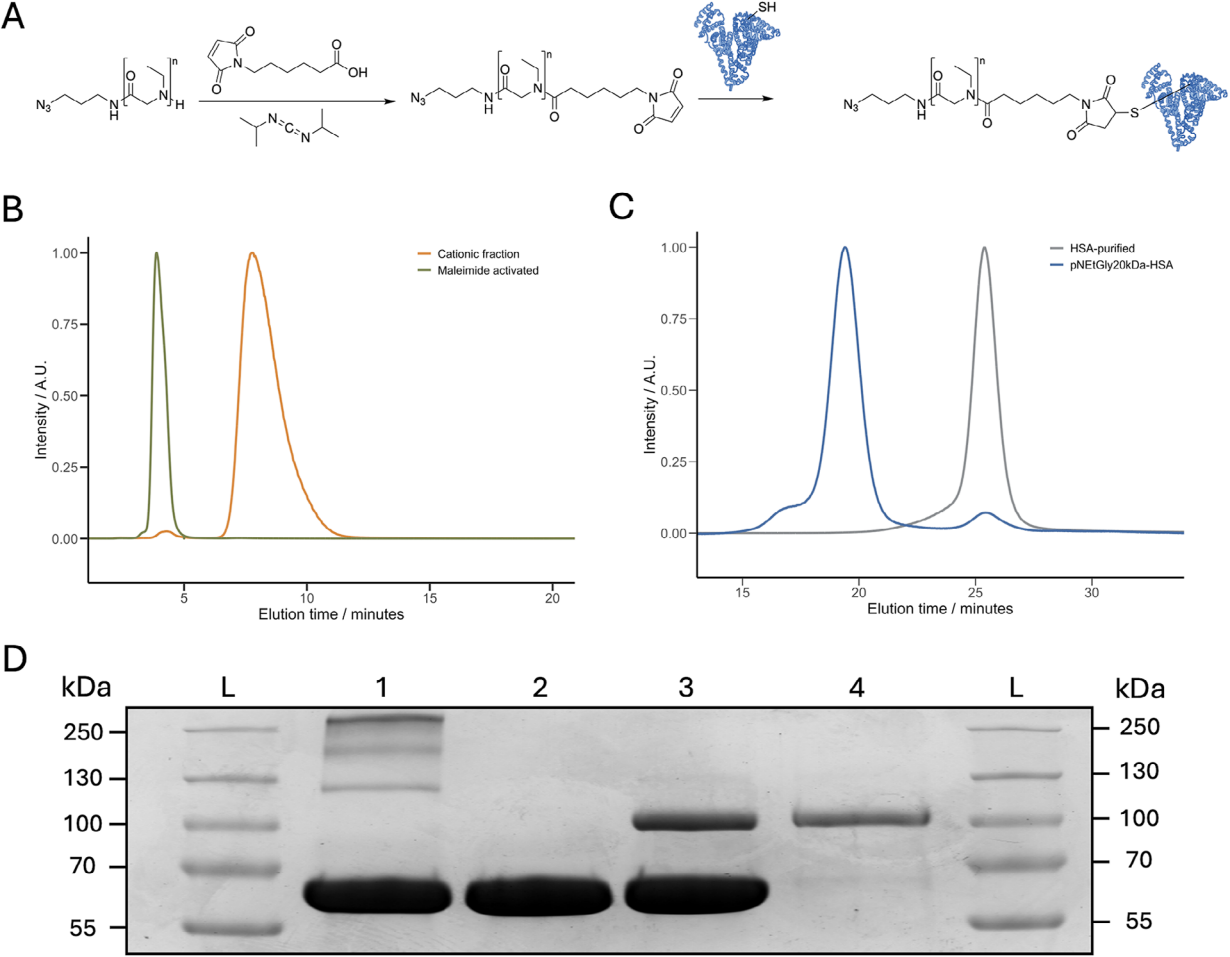
Overview of the conjugation of poly(*N‐*ethylglycine) to human serum albumine (HSA). (A) Functionalization of poly‐*N‐*Ethylglycine with 6‐Maleimidohexanoic acid to obtain maleimide functionalized ω end‐groups. (B) UV_220nm_ analytical cation‐exchange chromatography of the 21 kg mol^−1^ p*N*EtGly versus the maleimide functionalized polymer. (C) HPSEC UV_220nm_ analysis of the purified p*N*EtGly‐HSA conjugate versus the unmodified (purified) HSA protein. (D) SDS‐page analysis of 21 kg mol^−1^ polymer conjugation reactions of HSA. Lane 1, crude HSA; lane 2, purified HSA; lane 3, crude HSA conjugation product; lane 4, purified HSA‐p*N*EtGly conjugate.

In addition to lipid conjugates, polypeptoid‐based polymer–protein conjugates offer a versatile platform for improving protein‐based therapies. These conjugates rely on the conjugation of a single or multiple high molecular weight hydrophilic polymer (DP200‐600) to the protein [65, 66]. Pharmacokinetic properties of these conjugates are strongly influenced by the polymer molecular weight, and broad molecular weight distributions may therefore lead to increased variability among conjugates [67]. Therefore, it is highly beneficial that the polymers used for such applications have low dispersity values, enabling homogenous pharmacokinetic profiles for the final product. Similarly to the polymer‐lipid conjugates, ω end‐group fidelity is an important factor when functionalizing the polymers with the chemoselective handles that are necessary for functionalization. In this work, we report the conjugation of a *N*‐ethylglycine polymer (M_n_ of 21 kg mol^−1^, Ð = 1.04; characterized by HFIP GPC) with a model protein, namely human serum albumin (HSA) (Figure S18). HSA is a 66.4 kg mol^−1^ protein containing one free cysteine (Cys_34_) residue, allowing for thiol/maleimide conjugation [68]. Additionally, the isoelectric point of HSA is 4.7, and therefore this protein and the corresponding conjugate can be separated by using anion exchange chromatography. Upon conjugation, the increase in hydrodynamic radius and shielding of the electrostatic interactions between the protein and the ion‐exchange material can lead to increased retention, making HSA an ideal model for method development in polymer–protein conjugation [69]. Functionalization of the poly(*N*‐ethylglycine) was initiated by coupling 6‐maleimidohexanoic acid to the amine‐terminated polymer (>99% cationic fraction; characterized by CEC) (Figure 5A). Analytical cation‐exchange chromatography of the reaction mixture revealed a shift in elution toward an earlier retention time, consistent with successful introduction of the maleimide group onto the polymer ω end group (Figure 5B). The resulting maleimide‐functionalized p*N*EtGly polymers were subsequently reacted with human serum albumin (HSA) to form the HSA–polymer conjugate. The functional maleimide group can react with Cys_34_ to form the corresponding thioether bond, which ensures covalent attachment to the HSA protein. SDS‐PAGE analysis of the reaction mixture revealed a new protein band, as can be seen in lane 3 (Figure 5D). This band corresponds to the polymer–protein conjugate, confirming successful covalent attachment of the polymer to the protein. An excess of p*N*EtGly‐maleimide was used in the conjugation reaction; however, limited conversion is expected due to a combination of maleimide hydrolysis and the partial availability of free sulfhydryl groups, which are accessible on approximately 65%–70% of HSA molecules [69, 70, 71]. Separation of the unreacted HSA from the polymer–protein conjugate was performed via anion‐exchange chromatography. The first two UV_280nm_ absorbing peaks were collected and analyzed by SDS‐PAGE. Confirming its identity as the conjugated HSA fraction without any observable impurities (Figure 5D; Figure S19). High‐performance size exclusion chromatography (HPSEC) analysis on the peak fraction of the purified HSA–p*N*EtGly conjugate revealed a distinct shift to higher molecular weight, confirming successful covalent conjugation of the polymer to the HSA protein (Figure 5C). The minor shoulder observed in the HPSEC trace, indicative of HSA‐p*N*EtGly conjugate aggregates [69]. Detection at 220 nm showed 95% of the total peptide‐ and polymer‐containing material eluted as a single peak, confirming high overall purity (Figure 5C). Complementary analysis at 280 nm showed that 90% of the total HSA was present in the conjugated form, confirming efficient protein conjugation (Figure S20). These results demonstrate the successful synthesis of high molecular weight poly(*N*‐ethylglycine)–HSA conjugates with high conjugation efficiency and purity, highlighting the synthetic feasibility of p*N*EtGly for protein bioconjugation strategies.

## 3 Conclusions

In this work, we report an improved *N*‐ethylglycine NCA synthesis with a controlled polymerization approach that yields high molecular weight poly(*N*‐ethylglycine) up to DP of 400 with excellent molecular weight distributions (Ð<1.1) and quantitative heterotelechelic end‐group functionality. These properties are essential parameters for biomedical applications, as they enable increased homogenic pharmacokinetic profiles and increase efficiency and purity of their downstream applications. Given the synthetic potential outlined in the paper with the successful formation and purification of lipid– and protein–polymer conjugates, we highlight the applicability of p*N*EtGly in next‐generation drug delivery systems, specifically towards lipid‐based nanoparticles and polymer‐protein conjugates. Our findings highlight poly(*N*‐ethylglycine) as a relevant candidate for hydrophilic polymers in pharmaceutical applications.

## Conflicts of Interest

M.B. is named as inventor on the patent application Compounds and compositions for delivery of Agents to cells. 2022, P346117NL/JKR, which include poly(*N*‐ethyl‐glycine) lipid conjugates. The other authors declare no conflicts of interest.

## Supporting information




**Supporting File 1**: marc202500422‐sup‐0001‐SuppMat.docx.

## Data Availability

The data that support the findings of this study are available from the corresponding author upon reasonable request.
