# Supplementary material for: Synthesis of Heterotelechelic Poly(N‐ethylglycine) Polymers: from Polypeptoid Lipid Conjugates to Protein‐Polymer Conjugates
Source: Macromol Rapid Commun. 2025 Jul 16;46(20):e00422. doi: 10.1002/marc.202500422 (PMC12536396; doi:10.1002/marc.202500422)
Supplement: Supplementary file 1 — Supporting File 1: marc202500422‐sup‐0001‐SuppMat.docx. [file MARC-46-e00422-s001.docx]

Supporting information to:

**Synthesis of Heterotelechelic Poly(N-ethylglycine) Polymers: From Polypeptoid Lipid Conjugates to Protein-Polymer Conjugates**

Ewout Buijs^1^, Zlata Nagorna^1^, Joachim F. R. Van Guyse^1^, Matthias Barz^1^

^1^Leiden Academic Centre for Drug Research (LACDR), Leiden University, Einsteinweg 55, 2333 CC Leiden, The Netherlands

This file includes:

- Experimental procedures

- Figures S1–S20

- References

Total: 12 pages

7. Experimental Section

7.1 Materials

*Materials:* DCM was purchased from Honeywell and dried and distilled from CaH_2_ under nitrogen atmosphere. Hexane was purchased from Biosolve (Valkenswaard, The Netherlands). THF was purchased from Honeywell and distilled to remove the BHT stabilizer, subsequently it was transferred to Puresolve solvent purification system, from which it was collected. Hexafluoroisopropanol (HFIP) was purchased from Fluorochem (Hadfield Derbyshire, UK). Dimethylformamide (DMF) was purchased from Biosolve (Valkenswaard, The Netherlands). 3-chloropropan-1-amine hydrochloride was purchased from Synthonix. Sodium Azide was purchased from Sigma-Aldrich (Zwijndrecht, The Netherlands). Glyoxylic acid solution 50% in water was purchased from VWR (Amsterdam, The Netherlands). Ethylamine solution 66-72.0% in water was purchased from Sigma-Aldrich Hydrochloric acid 37% was purchased from Carl Roth (Karlsruhe, Germany). Sodium Hydroxide was purchased from Sigma-Aldrich. Di-tert-butyl-carbonate was purchased from Sigma-Aldrich. Thionyl Chloride (99.7%) was purchased from Fisher Scientific (Leiden, The Netherlands). CM Sephadex C-50 was purchased from GE Healthcare. Ammonium Hydroxide solution 25% was purchased from Fisher Scientific. N,N'-Diisopropylcarbodiimide (DIC) was purchased from Tokyo Chemical Industries (TCI) (Liechtenstein, Switzerland). Palmitic acid was purchased from Sigma-Aldrich. HSA was purchased from PAN-Biotech (Aidenbach, Germany). Deuterated solvents were purchased from Deutero GmbH (Kastellaun, Germany). Sodium Chloride was purchased from Sigma-Aldrich. Potassium hydroxide was purchased from Sigma-Aldrich. Sodium phosphate was purchased from Fluka/Thermofisher (Zwijndrecht, The Netherlands). Sodium acetate was purchased from Fluka/thermofisher. Tris base was purchased from Sigma-Aldrich.

^1^H NMR spectra were acquired using a Bruker AC 400 spectrometer (Billerica, MA, USA) operating at 400 MHz. Two-dimensional ^1^H DOSY NMR spectra were recorded on a Bruker Avance III HD 400 spectrometer operating at 400MHz for ^1^H measurements and 100MHz for ^13^C measurements. All ^1^H and ^13^C spectra were obtained at room temperature and calibrated based on solvent signals. Fourier-transform infrared (FT-IR) spectroscopy was conducted using a JASCO FT-IR 4600 spectrometer. Samples were measured either as neat films or in solution. Gel permeation chromatography (GPC) was performed on two separate SEC systems. The first system uses HFIP supplemented with 3 g·L−1 potassium trifluoroacetate (KTFA) as the eluent at 40°C with a flow rate of 1 mL·min^−1^ The columns were packed with modified silica (pore size: 10, 30 and 400 nm, PFG column particle size: 7 µm, porosity: 100-100.000, 1000-300.000 and 10.000-1.000.000 Å respectively). Polymethylmethacrylate (PMMA) standards (Polymer Standards Services GmbH, Mainz, Germany) were used for calibration, with toluene as the internal standard. While PMMA standards were used to determine the dispersity (Đ) values, more accurate number-average molecular weights (Mn) were obtained using polysarcosine (pSar) standards reported by Weber et al. (Macromolecules, 2018). ^1^ These pSar standards were characterized by static light scattering, yielding molecular weight values that more accurately reflect the true values of the polymers analyzed. A refractive index detector (Jasco (Gross-Umstadt, Germany) RI-4030) and an UV-VIS detector (at 230nm unless otherwise stated; Jasco (Gross-Umstadt, Germany) UV-4070) were used for polymer detection in HFIP. The second system uses DMF supplemented with 10mM LiCl_4_ as the eluent at 40°C with a flow rate of 0.8 mL·min^−1^. The columns were packed with modified silica (pore size: 10, 30 and 400 nm, PFG column particle size: 7 µm, porosity: 100-100.000, 1000-300.000 Å respectively). PEG standards (Supplier (City, Country) were used for calibration. Polymers were detected by refractive index detector (Jasco (Gross-Umstadt, Germany) RI-4030). Analytical ion-exchange chromatography was conducted using a TSKgel® SP-5PW HPLC column, a strong cation exchanger. Phosphate buffers at concentrations of 0.5 mM and 2 mM with a flow rate of 0.5ml/min. The polymers were detected by UV-VIS (at 220nm unless stated otherwise). UPLC analysis was performed on a WATERS ACQUITY UPLC system loaded with a BEH C18 1.7µm 2.1 x 50 mm column. The mobile phases consisted of eluent A (Milli-Q water with 0.1% trifluoroacetic acid), eluent C (methanol containing 5% Milli-Q water), and eluent D (isopropanol). The flow rate was set to 0.4 mL/min with the following gradient program: the run was initiated with 95% A and 5% C, which was maintained for 3.0 minutes. From 3.0 to 4.0 minutes, the composition was linearly changed to 50% A and 50% C. Between 4.0 and 6.5 minutes, the mobile phase was shifted to 100% C. From 6.5 to 8.0 minutes, the composition was adjusted to 80% C and 20% D, followed by a transition to 20% C and 20% D between 8.0 and 8.6 minutes. The system was then re-equilibrated to the initial condition of 95% A and 5% C from 8.6 to 11.0 minutes. High-performance size exclusion chromatography was performed using a Superdex™ 200 10/300 GL column. The mobile phase consisted of 50 mM phosphate buffer supplemented with 200 mM NaCl. The chromatography was carried out at a flow rate of 0.5 mL/min, and elution was monitored by UV-Vis detection at 220 nm and 280 nm for 60 minutes. SDS-PAGE analysis was performed using 10% polyacrylamide gels. Electrophoresis was initiated at 70 V for 15 minutes to allow sample entry into the resolving gel, after which the voltage was increased to 120 V and maintained until the dye front migrated to approximately 1 cm from the bottom of the gel. Gels were stained with Coomassie R-250 and for 1h and subsequently destained with a destaining solution (50% methanol, 10% acetic acid and 40% water) until no visible background signal was observed and subsequently visualized using a Bio-Rad ChemiDoc imaging system.

7.2 Methods

*Synthesis of 3-Azido-1-propanamine:* The synthesis of 3-azido-1-propanamine was performed according to previously reported procedure.^2^ 3-chloropropylamine hydrochloride (4 g, 30.77 mmol) and sodium azide (6 g, 92.30 mmol) were dissolved in 30 mL of Milli-Q water and stirred overnight at 80 °C. Subsequently, around 70% of the water was removed under reduced pressure. The solution was cooled on ice and 50 mL of diethyl ether and KOH (6 g) were added. The mixture was allowed to phase separate, and the organic phase was collected. The aqueous phase was extracted two more times with diethyl ether (20 mL) and added to the initial organic phase. The organic phase was dried over Na_2_SO_4_ and subsequently concentrated under reduced pressure to obtain a very slightly yellowish oil. The crude product purified by distillation at a pressure of 10 mbar and 60°C to obtain the final product appearing as a colorless slightly viscous liquid (1721 mg, 14,79 mmol, 56%). ^1^H NMR (400 MHz, CDCl_3_ δ): 1.15 (s, 2H, *H2N-*), 1.66 (q, 2H, -CH2-*CH2*-CH2-), 2.73 (t, 2H, H2N-*CH2*-), 3.30 ppm (t, 2H, N3-*CH2*-).

*Synthesis of N-ethylglycine • HCl:* The synthesis of N-ethylglycine • HCl was carried out according to previously reported procedure with slight modifications.^3^ A 60% aqueous solution of ethylamine (90 mL, 1.13 mol) and a 50% aqueous solution of glyoxylic acid (167.5 mL, 2.26 mol) were added to 1 L of Milli-Q water and stirred at room temperature for 24 hours. Concentrated hydrochloric acid (460 mL) was then added, and the reaction mixture was refluxed for 12 hours. Following the reaction, water was removed under reduced pressure to yield a dark brown solid. The crude product was purified by triple recrystallization from a 2:1 methanol:diethyl ether mixture, affording N-ethylglycine as a white solid (54 g, 0.38 mol, 34%). ^1^H NMR (400 MHz, CDCl_3_ δ): 1.19 (t, 3H, *CH3*-CH2-), 2.93 (q, 2H, CH3-*CH2*-NH-), 3.82 (s, 2H, RN-*CH2*-CR), 9.24 (br, 2H, NH-HCl), 13.74 (br, 1H, -COOH). ^13^C NMR (100 MHz, CDCl_3_ δ): 10.78 (*CH3*-CH2-), 41.88 (CH3-*CH2*-), 46.36 (RN-*CH2*-CR), 168.15 (COOH).

*Synthesis of Boc-N-Ethylglycine:* The synthesis of Boc-N-ethylglycine was carried out according to previously reported procedure with slight modifciations.^3^ To a solution of sodium hydroxide (5.7 g, 0.143 mol) in a 75 mL mixture of Milli-Q water and isopropyl alcohol, N-ethylglycine·HCl (10 g, 71.6 mmol) was added. Di-tert-butyl dicarbonate (15.6 g, 71.6 mmol) was then added portion-wise. The reaction mixture was stirred at room temperature for 2 hours, followed by refluxing for 30 minutes at 95°C. After cooling to room temperature, the mixture was washed with 100 mL of pentane. The aqueous layer was acidified to pH 1–2 using concentrated HCl (37%) and extracted three times with 100 mL of diethyl ether. The combined organic phases were concentrated under reduced pressure to yield a slightly yellow oil, which was dried under high vacuum (<1 mbar) at 40 °C to afford a white, chunky solid. The solid was crushed to a fine white powder using a mortar and pestle of the product (12.1 g, 78%). ^1^H NMR (400 MHz, CDCl_3_ δ): 1.15 (t, 3H, *CH3*-CH2-), 1.46 (d, 9H, *C3H9-*), 2.93 (q, 2H, CH3-*CH2*-NH-), 3.31 (m, 2H, COOH-*CH2*-), 9.10 (br s, 1H, COOH-).

*Synthesis of N-Ethylglycine N-carboxyanhydride (NCA):* The synthesis of *N*-ethylglycine N-carboxyanhydride was adapted from previously reported procedures of N-alkylated N-carboxyanhydrides with various modifications.^4,5^ Boc-*N*-ethylglycine (10.3 g, 50.7 mmol) was dissolved in anhydrous tetrahydrofuran (THF) to a final concentration of 0.5 g/mL. The solution was cooled in an ice bath to 0°C, and thionyl chloride (7.2 g, 60.8 mmol) was added dropwise over 60 minutes under continuous stirring. Following the addition, the reaction mixture was stirred at room temperature for 2-3 hours. Complete conversion of the starting material was confirmed by ^1^H NMR spectroscopy. Excess THF was removed by distillation under reduced pressure in an inert atmosphere at 35 °C and 100 mbar. The resulting residue was washed five times with 100 mL of dry n-hexane under a stream of dry nitrogen. Residual hexane was removed by distillation under reduced pressure in an inert atmosphere at 35 °C and 100 mbar. The remaining residue was dissolved in pre-dried dichloromethane (DCM) and filtered through a Celite 513 pad under a nitrogen atmosphere. The solvent was evaporated under reduced pressure to yield the crude NCA as a slightly yellow liquid (5.53 g, 42.8 mmol, 84%). For purification, the crude product was distilled under high vacuum (<0.2 mbar) at an oil bath temperature of 130 °C. The final product was obtained as a colorless liquid (1.2 g, 19%). ^1^H NMR (400 MHz, CDCl_3_ δ): 1.25 (t, 3H, *CH3*-CH2-), 3.50 (q, 2H, CH3-*CH2*-), 4.11 (s, 2H, CO-*CH2*-NR-). ^13^C NMR (100 MHz, CDCl_3_ δ): 12.62 (*CH3*-CH2-), 38.61 (CH3-*CH2*-), 48.48 (C4), 151.91 (C2), 165.69 (C5).

*Synthesis of poly-(N-ethylglycine):* The conditions for the synthesis of poly-(*N*-ethylglycine) have been adapted from previously published work.^6,7^ *N*-Ethylglycine-NCA was transferred into a pre-dried Schlenk tube under a dry nitrogen atmosphere and dissolved in pre-dried dichloromethane (DCM) at a concentration of 400mM. Acetic acid was then added to the reaction mixture at an initial [Initiator]₀/[Acetic acid]₀ ratio of 1:5. Polymerization was initiated by the addition of 3-azido-1-propanamine at the desired monomer-to-initiator ([M]₀/[I]₀) ratio. The reaction was stirred at room temperature, and its progress was monitored by IR spectroscopy. Completion was confirmed by the disappearance of the characteristic NCA absorption peaks at 1858 and 1788 cm⁻¹. The resulting polymer was precipitated in diethyl ether and collected by centrifugation at 5000 rpm for 10 minutes. This precipitation and centrifugation step was repeated twice. The final product was obtained by vacuum drying of the pellet. ^1^H NMR (400 MHz, CDCl_3_ δ): 1.00 (m, 3**x*H, *CH3*-CH2-), 1.65 (q, 2H, N3-CH2-*CH2*-CH2-N-), 3.30 (m, 2**x*H, CH3-*CH2*-), 4.16 (m, 2**x*H, CO-*CH2*-), *x* = Dp. ^13^C NMR (100 MHz, CDCl_3_ δ): 12.49 (*CH3*-CH2-), 42.26 (CH3-*CH2*-), 47.70 (RN-*CH2*-CR), 168.98 (CO).

*Purification of poly-(N-ethylglycine) via cation exchange:* Crude poly-N-ethylglycine was purified using an NCG BioRad system equipped with a Toyopearl SP650M cation exchange column. Neutral components were removed by washing the column with Milli-Q water until no absorbance at 220 nm was detected. The cationic fraction was then eluted using a 174-fold dilution of a 25% ammonia solution, with elution monitored by UV absorbance at 220 nm. Ammonia was subsequently removed under reduced pressure, and the eluate was concentrated. The resulting solution was transferred to a 50 mL Falcon tube and lyophilized to yield the purified polymer as a white, fluffy powder.

*Synthesis of poly-N-(ethylglycine)-palmitamide:* Poly-*N*-(ethylglycine) (25 mg, 0.015 mmol) was dissolved in dry dichloromethane (DCM) at a concentration of 50 mg/mL under a dry nitrogen atmosphere. Palmitic acid (38 mg, 0.15 mmol) was added, and the mixture was stirred for 5 minutes until fully dissolved. N,N′-Diisopropylcarbodiimide (DIC, 23 µL, 0.15 mmol) was then added, and the reaction was stirred for 1 hour at room temperature. The reaction mixture was precipitated in diethyl ether and centrifuged at 5000 rpm for 10 minutes. The resulting pellet was redissolved in DCM, and the precipitation step was repeated twice. The final pellet was dissolved in Milli-Q water, dialyzed using a 1 kDa molecular weight cut-off (MWCO) membrane against fresh Milli-Q water. Subsequently the product was lyophilized to yield the palmitoylated polymer as a white, fluffy powder (yield; 60%, 15 mg, 0.009 mmol). ^1^H NMR (400 MHz, CDCl_3_ δ): 0.85 (t, 3H, CH3-), 0.98 (m, 98H, CH3-), 1.23 (bd, 37H, -CH2-), 1.65 (q, 2H, N3-CH2-*CH2*-CH2-NH-), 2.01 (t, 2H, N3-*CH2*-), 3.35 (m, 117H, CH3-*CH2*-), 4.16 (br, 60H, CO-*CH2*-).

*Purification of HSA:* HSA protein was loaded onto a DEAE anion exchange column pre-equilibrated with 20 mM Tris buffer, pH 6.95. The column was washed with 2 column volumes (CV) of the same buffer to remove unbound impurities, and HSA was eluted using a 20 CV linear gradient toward 20 mM Tris buffer containing 200 mM NaCl, pH 6.95. Elution was monitored by UV absorbance at 220 nm and 280 nm, with fractions collected every 10 mL. Fractions containing a single band of HSA, as determined by SDS-PAGE, were pooled and concentrated using an ultrafiltration system equipped with a 2 kDa molecular weight cut-off (MWCO) membrane. The concentrated HSA was then buffer exchanged into 20 mM sodium acetate buffer, pH 5.5, by repeated cycles of concentration followed by dilution with fresh sodium acetate buffer; this process was repeated three times.

*Synthesis of poly(N*-*ethylglycine)-HSA conjugates:* The coupling conditions have been adapted from previously published work.^8^ Poly(N-ethylglycine)-maleimide (12 mg, 0.0006 mmol) was added to a solution of pre-purified HSA (20 mg, 0.0003 mmol) in 20 mM sodium acetate buffer, pH 5.5, at a final protein concentration of 2 mg/mL. The reaction mixture was stirred at room temperature overnight. The resulting crude protein–polymer conjugate was analyzed by SDS-PAGE to verify conjugation.

*Purification of poly(N*-*ethylglycine)-HSA conjugates:* The crude protein–polymer mixture was loaded onto a DEAE anion exchange column equilibrated with 20 mM Tris buffer at pH 6.95. The column was washed with 2 column volumes (CV) of the same buffer to remove excess polymer–maleimide. Elution of the conjugate was performed using a 50 CV gradient from 20 mM Tris buffer to 20 mM Tris buffer containing 200 mM NaCl, both at pH 6.95. Elution was monitored by UV absorbance at 220 nm and 280 nm, with fractions collected every 10 mL. Fractions were analyzed by SDS-PAGE, and those containing the polymer-protein conjugate were pooled and concentrated using a 10,000 kDa molecular weight cut-off (MWCO) spin filter.

7.3 Supporting figures


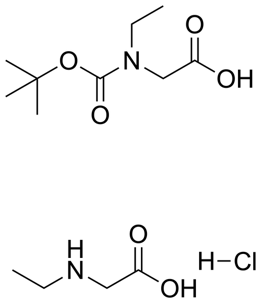


**Supporting Figure 1:** ^1^H NMR of N-ethylglycine hydrochloride.


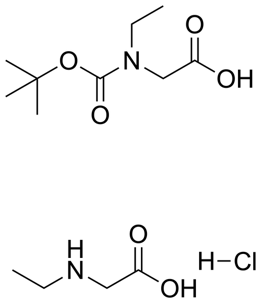


**Supporting Figure 2:** APT ^13^C NMR of N-ethylglycine hydrochloride.

**Supporting Figure 3:** ^1^H NMR of Cbz-N-ethylglycine.


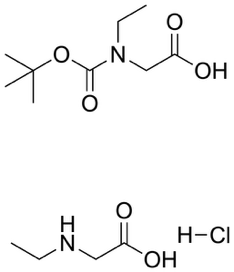


**Supporting Figure 4:** ^1^H NMR of Boc-N-ethylglycine.


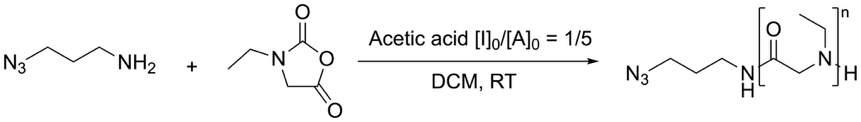


**Supporting Figure 5:** ^1^H NMR of N-ethylglycine N-carboxy anhydride, synthesized via Cbz protection group and acetyl chloride / acetic anhydride.


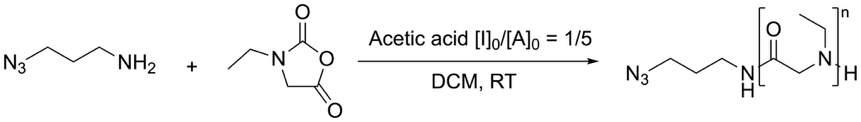


**Supporting Figure 6:** ^1^H NMR of N-ethylglycine N-carboxyanhydride, synthesized via Boc protection group and thionyl chloride pre-distillation.


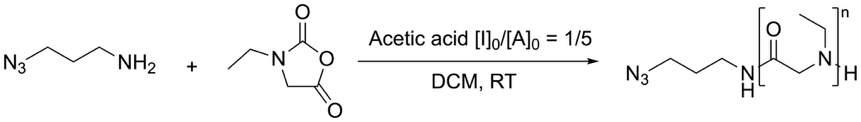


**Supporting Figure 7:** ^1^H NMR of N-ethylglycine N-carboxyanhydride, synthesized via Boc protection group and thionyl chloride post-distillation.


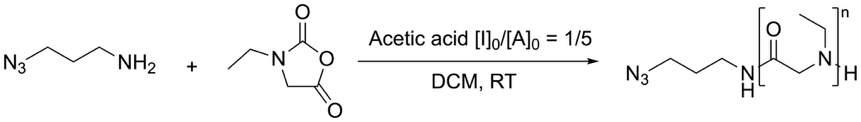
 **Supporting Figure 8:** APT ^13^C NMR of N-ethylglcyine N-carboxyanhydride, synthesized via Boc protection group and thionyl chloride post-distillation.


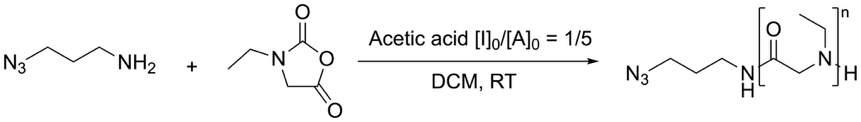


**Supporting Figure 9:** ^1^H NMR of 3-azido-propyl-amine.


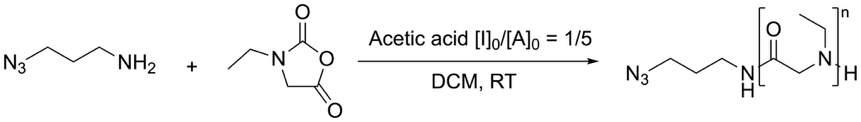


**Supporting Figure 10:** ^1^H NMR of poly(*N*-ethylglycine) ([M]_0_/[I]_0_/[AA]_0_ ; 100/1/5)


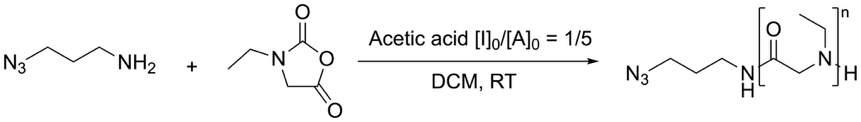


**Supporting Figure 11:** APT ^13^C NMR of poly(*N*-ethylglycine) ([M]_0_/[I]_0_/[AA]_0_ ; 100/1/5)


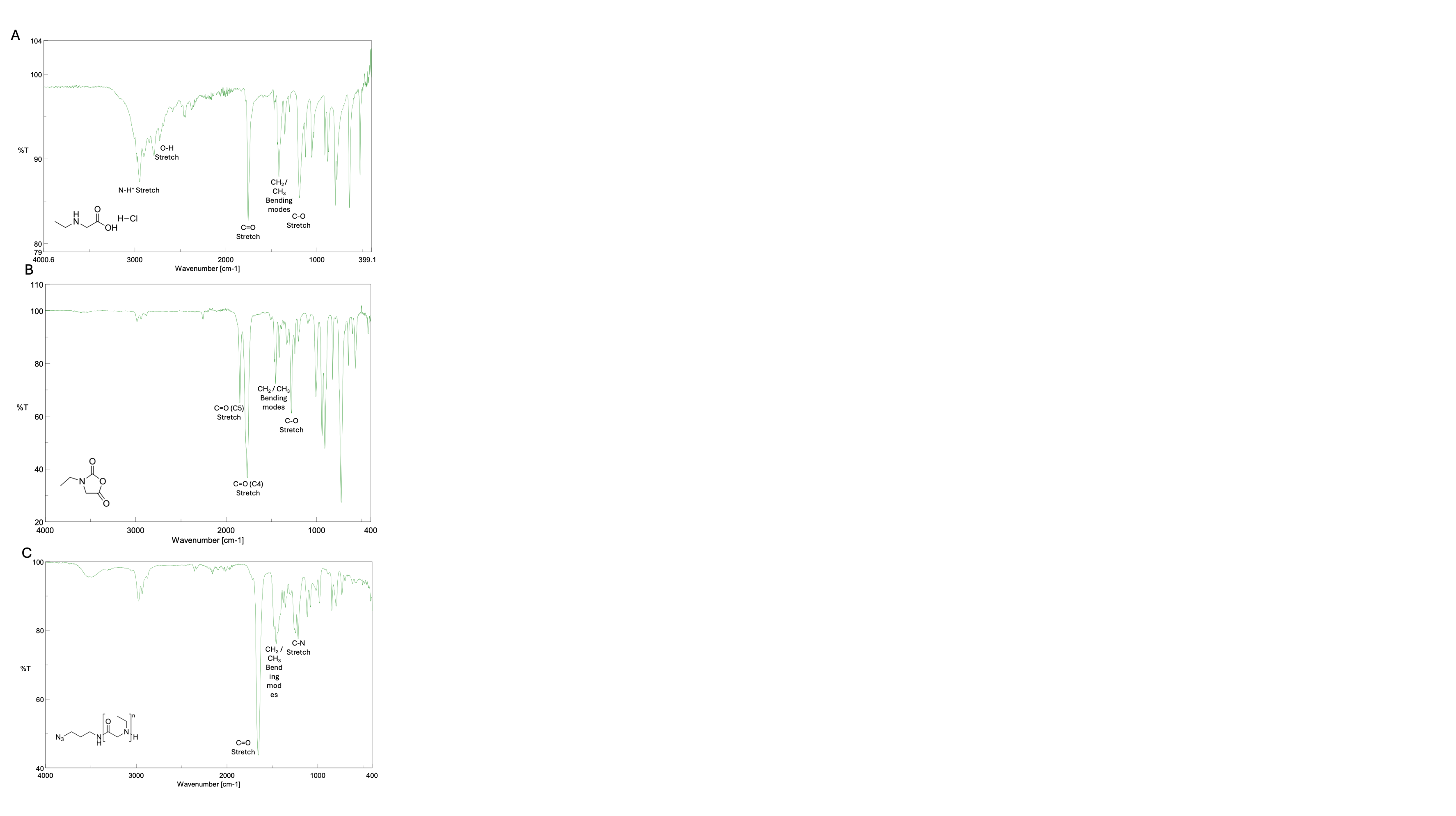


**Supporting Figure 12:** FT-IR spectra of *N*-ethylglycine and its derivatives, with characteristic peaks assigned **A)** *N*-Ethylglycine HCl **B)** *N*-Ethylglycine N-carboxyanhydride **C)** poly(*N*-ethylglycine) ([M]_0_/[I]_0_/[AA]_0_ ; 100/1/5)


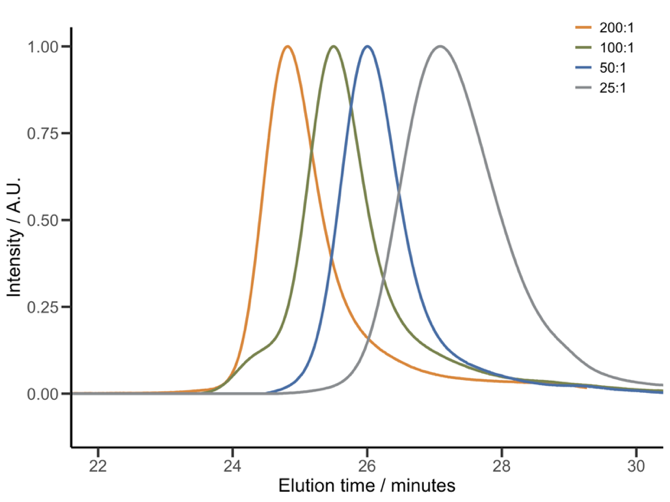


**Supporting Figure 13:** HFIP SEC traces of non-catalyzed poly(*N*-ethylglycine) for various degrees of polymerizations in accordance with Table 1.

**
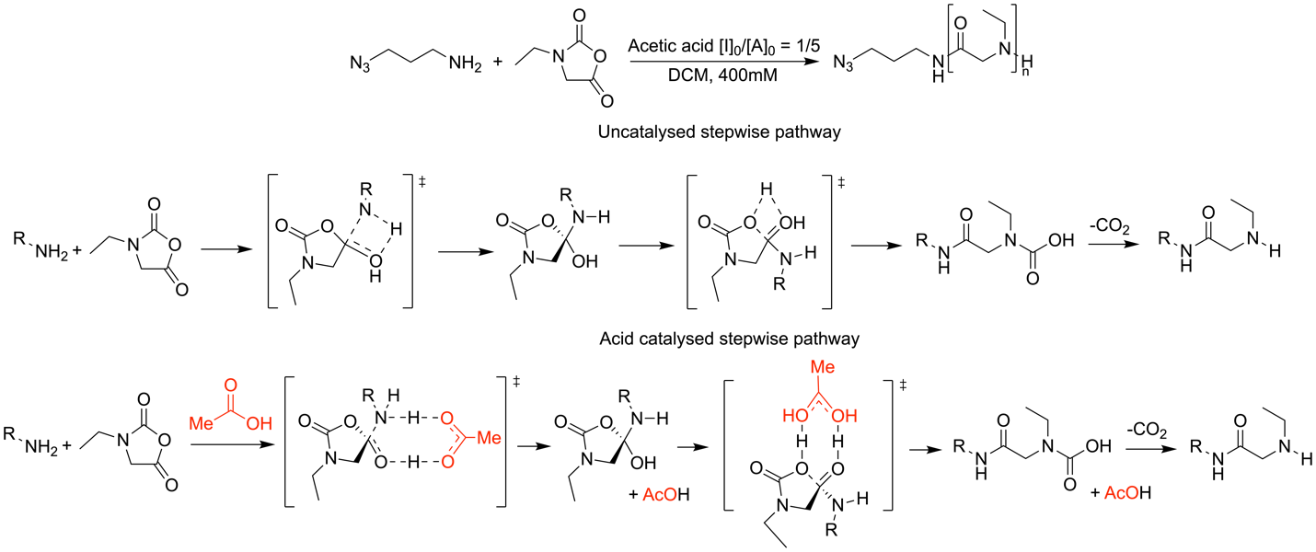
 Supporting Figure 14:** Schematics of the uncatalyzed and catalyzed stepwise pathways for *N*-ethylglycine polymerization.

**Supporting Figure 15:** ^1^H NMR of palmitic acid pNEtGly polymer conjugate.

**Supporting Figure 16:** DOSY ^1^H NMR of palmitic acid pNEtGly polymer conjugate.

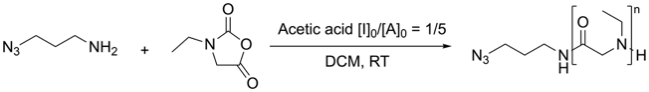


**Supporting Figure 17:** ^1^H NMR of poly(*N*-ethylglycine) (20kDa) before (top) and after (bottom) addition of the maleimide end-group.


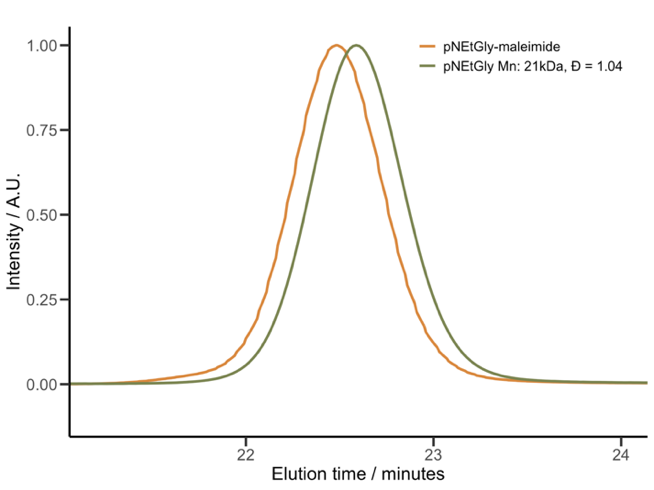


**Supporting figure 18:** HFIP GPC of 20kDa pNEtGly for protein conjugation. **A)** UV_220nm_ HFIP SEC plots of amine terminal 20kDa poly(*N*-ethylglycine). **B)** UV_220nm_ HFIP SEC plots of maleimide terminal poly(*N*-ethylglycine).


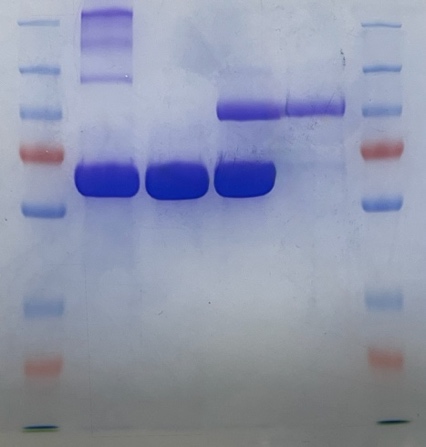

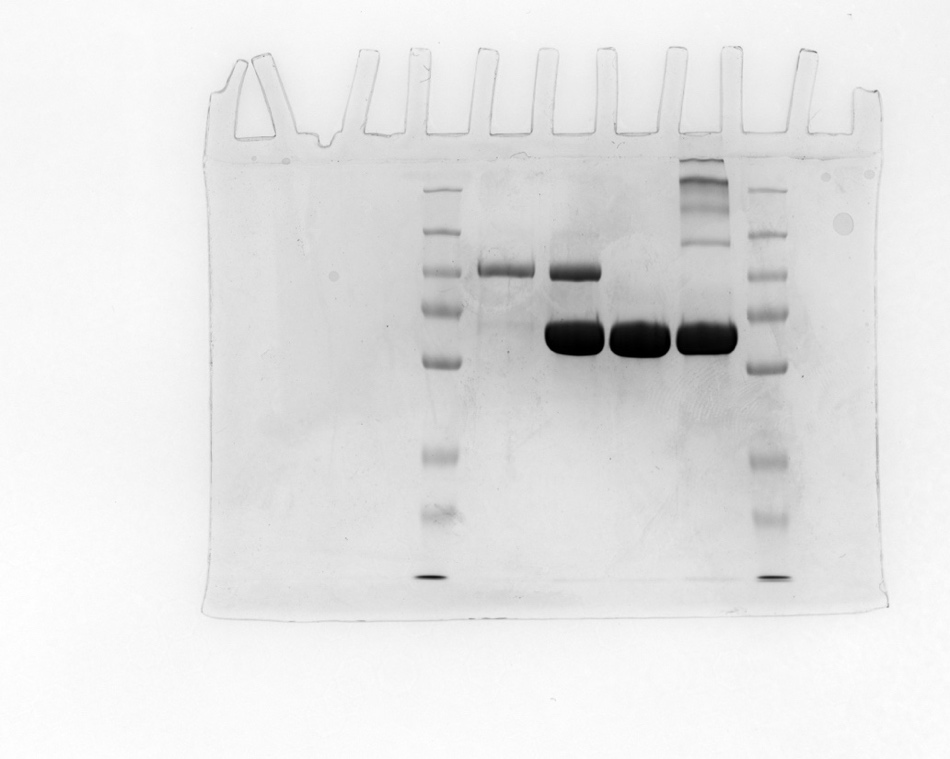

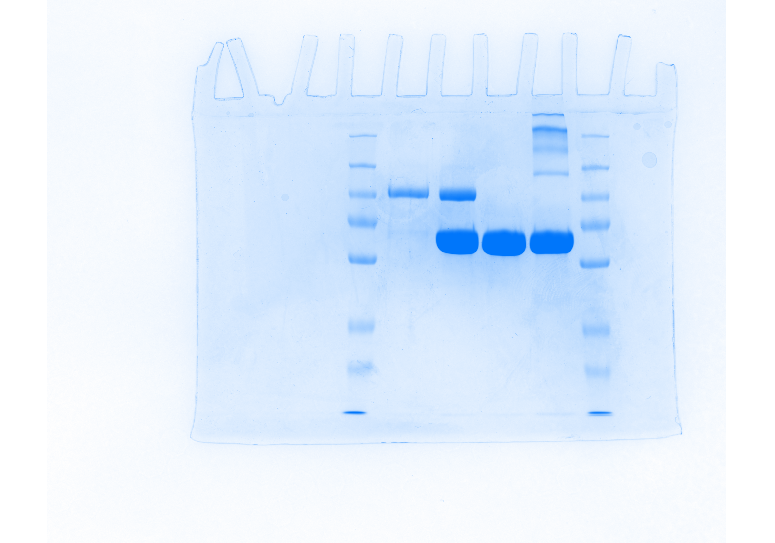


**Supporting Figure 19:** Full SDS-page analysis of 20 kDa polymer conjugation reactions of HSA in color, black/white and blue. Lane 1, Protein ladder; lane 2, crude HSA; lane 3, purified HSA; lane 4, crude HSA conjugation product; lane 4, purified HSA-pNEtGly conjugate; lane 5, Protein ladder.


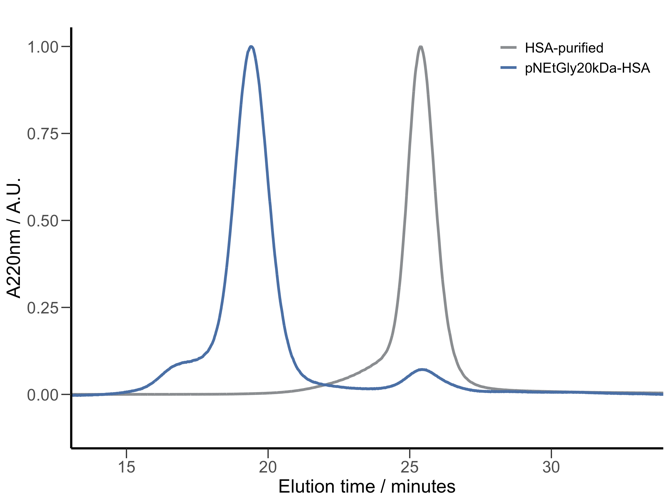

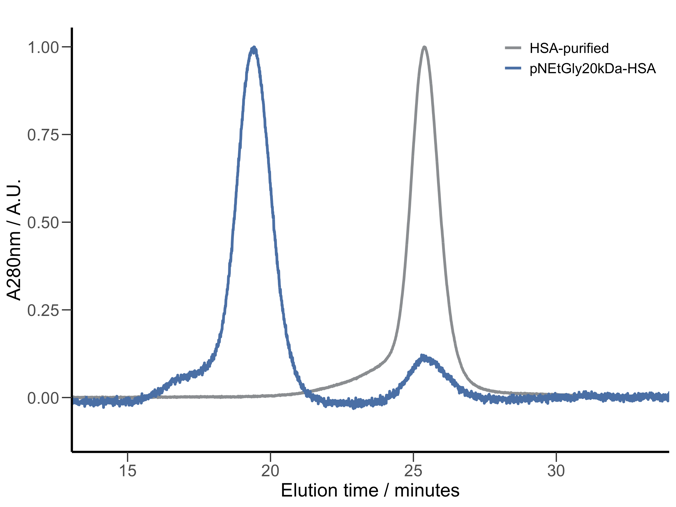


**Supporting Figure 20:** HPSEC UV_220nm_ & UV_280nm_ analysis of the purified pNEtGly20kDA-HSA conjugate versus the unmodified HSA protein.

References

(1) Weber, B.; Birke, A.; Fischer, K.; Schmidt, M.; Barz, M. Solution Properties of Polysarcosine: From Absolute and Relative Molar Mass Determinations to Complement Activation. *Macromolecules* **2018**, *51* (7), 2653–2661. https://doi.org/10.1021/acs.macromol.8b00258.

(2) Shi, Y.; Pierce, J. G. Synthesis of the 5,6-Dihydroxymorpholin-3-One Fragment of Monanchocidin A. *Org. Lett.* **2015**, *17* (4), 968–971. https://doi.org/10.1021/acs.orglett.5b00069.

(3) Fetsch, C.; Grossmann, A.; Holz, L.; Nawroth, J. F.; Luxenhofer, R. Polypeptoids from N-Substituted Glycine N-Carboxyanhydrides: Hydrophilic, Hydrophobic, and Amphiphilic Polymers with Poisson Distribution. *Macromolecules* **2011**, *44* (17), 6746–6758. https://doi.org/10.1021/ma201015y.

(4) Salas-Ambrosio, P.; Tronnet, A.; Badreldin, M.; Ji, S.; Lecommandoux, S.; Harrisson, S.; Verhaeghe, P.; Bonduelle, C. Effect of N-Alkylation in N-Carboxyanhydride (NCA) Ring-Opening Polymerization Kinetics. *Polym. Chem.* **2022**, *13* (43), 6149–6161. https://doi.org/10.1039/D2PY00985D.

(5) Sisido, M.; Imanishi, Y.; Okamura, S. Polymerization of amino acid derivatives by polymer catalysts. III. Chain effect polymerization induced by poly(N-ethylglycine) diethylamide. *Biopolymers* **1969**, *7* (6), 937–947. https://doi.org/10.1002/bip.1969.360070609.

(6) Wang, S.; Lu, M.-Y.; Wan, S.-K.; Lyu, C.-Y.; Tian, Z.-Y.; Liu, K.; Lu, H. Carboxylic Acid-Catalyzed Controlled Ring-Opening Polymerization of Sarcosine N-Carboxyanhydride: Fast Kinetics, Ultra-High Molecular Weight, and Mechanistic Insights. Chemistry October 5, 2023. https://doi.org/10.26434/chemrxiv-2023-v7ksw.

(7) Nagorna, Z.; Barz, M.; Van Guyse, J. F. R. Toward Quantitative End-Group Fidelity in the Synthesis of High Molecular Weight Polysarcosine. *ACS Macro Lett.* **2025**, 532–537. https://doi.org/10.1021/acsmacrolett.5c00165.

(8) Pabst, T. M.; Buckley, J. J.; Ramasubramanyan, N.; Hunter, A. K. Comparison of Strong Anion-Exchangers for the Purification of a PEGylated Protein. *J. Chromatogr. A* **2007**, *1147* (2), 172–182. https://doi.org/10.1016/j.chroma.2007.02.051.
